# Prevention of downhill walking-induced muscle damage by non-damaging downhill walking

**DOI:** 10.1371/journal.pone.0173909

**Published:** 2017-03-13

**Authors:** Sumiaki Maeo, Masayoshi Yamamoto, Hiroaki Kanehisa, Kazunori Nosaka

**Affiliations:** 1 Faculty of Sport Sciences, Waseda University, Tokorozawa, Saitama, Japan; 2 Research Fellow of Japan Society for the Promotion of Science, Chiyoda, Tokyo, Japan; 3 Sports and Life Science, National Institute of Fitness and Sports in Kanoya, Kanoya, Kagoshima, Japan; 4 School of Medical and Health Sciences, Edith Cowan University, Joondalup, Western Australia, Australia; West Virginia University School of Medicine, UNITED STATES

## Abstract

**Purpose:**

Mountain trekking involves level, uphill, and downhill walking (DW). Prolonged DW induces damage to leg muscles, reducing force generating ability and muscle coordination. These increase risks for more serious injuries and accidents in mountain trekking, thus a strategy to minimize muscle damage is warranted. It has been shown that low-intensity eccentric contractions confer protective effect on muscle damage induced by high-intensity eccentric contractions. This study tested the hypothesis that 5-min non-damaging DW would attenuate muscle damage induced by 40-min DW, but 5-min level walking (LW) would not.

**Methods:**

Untrained young men were allocated (n = 12/group) to either a control or one of the two preconditioning groups (PRE-DW or PRE-LW). The PRE-DW and PRE-LW groups performed 5-min DW (-28%) and 5-min LW, respectively, at 5 km/h with a load of 10% body mass, 1 week before 40-min DW (-28%, 5 km/h, 10% load). The control group performed 40-min DW only. Maximal knee extension strength, plasma creatine kinase (CK) activity, and muscle soreness (0–100 mm visual analogue scale) were measured before and 24 h after 5-min DW and 5-min LW, and before and 24, 48, and 72 h after 40-min DW.

**Results:**

No significant changes in any variables were evident after 5-min DW and 5-min LW. After 40-min DW, the control and PRE-LW groups showed significant (*P*<0.05) changes in the variables without significant differences between groups (control vs. PRE-LW; peak strength reduction: -19.2 ± 6.9% vs. -18.7 ± 11.0%, peak CK: 635.5 ± 306.0 vs. 639.6 ± 405.4 U/L, peak soreness: 81.4 ± 14.8 vs. 72.0 ± 29.2 mm). These changes were significantly (*P*<0.05) attenuated (47–64%) for the PRE-DW group (-9.9 ± 9.6%, 339.3 ± 148.4 U/L, 27.8 ± 16.8 mm).

**Conclusions:**

The results supported the hypothesis and suggest that performing small volume of downhill walking is crucial in preparation for trekking.

## Introduction

Walking in mountain areas such as trekking is a popular physical activity among many people including seniors around the world [1, 2]. Mountain trekking involves level, uphill, and downhill walking (DW) on uneven and rugged terrain, giving challenges especially for those who are novice or who do it infrequently [3]. While physical and mental health benefits produced by mountain trekking are widely recognized [1, 3], there are high risks of accidents such as falling or slipping during mountain trekking, which could result in serious injuries or death in the worst case [1, 2].

During DW, lower limb muscles especially the knee extensors predominantly perform eccentric contractions, where the force is produced while the muscle-tendon complex is lengthened to control a walking speed and/or to absorb shocks [4]. Exercises mainly consisting of eccentric contractions induce muscle damage, presumably due to a high strain on weak sarcomeres and/or extra cellular matrix surrounding muscle fibers [5], resulting in micro injuries to contractile proteins such as Z-line and A-band [6]. Muscle damage is typically represented by decreased muscle function, delayed onset muscle soreness (DOMS), and increased plasma creatine kinase (CK) activity lasting for several days after exercise, when they are performed for the first time or with a long interval from a previous bout [7]. Previous studies have shown that DW as well as mountain trekking induces muscle damage that requires several days to recover [3, 8]. Falling-related accidents and fatalities during mountain trekking are at least in part attributable to DW-induced muscle damage, in combination with accumulating fatigue from uphill and level walking [1, 2]. Even if accidents are avoided during mountain trekking, muscle damage could affect daily activities for several days, and could increase the risk of injuries [9]. Therefore, it is of importance to develop an intervention that minimizes DW-induced muscle damage, especially for beginners.

The magnitude of muscle damage is largely reduced when the same or similar exercise is repeated within several weeks, which is known as the repeated bout effect [10]. Burt et al. [11] reported that lower‐volume muscle‐damaging exercise (5 sets of 10 squats, 80% of body mass load) protected against high‐volume muscle‐damaging exercise (10 sets of 10 squats, 80% of body mass load) performed 2 weeks later. We [12] showed that changes in indirect markers of muscle damage (DOMS, decreases of maximal knee extension torque, increases in CK activity) after 40-min DW were significantly reduced, when a bout of 20-min DW that induced only small changes in these markers was performed 1 week before the 40-min DW. It should be noted that muscle damage was induced by the initial exercise bout in these studies.

It does not appear that muscle damage is a prerequisite for muscle damage protection to be conferred. In fact, using a single-joint eccentric exercise model such as elbow flexion or knee extension eccentric contractions, previous studies showed that even non-damaging low-intensity eccentric contractions performed within 2 weeks prior to maximal eccentric contractions reduced the magnitude of muscle damage after damaging high-intensity eccentric exercise [13, 14]. Thus, it is assumed that even shorter-duration DW, which does not induce muscle damage, may still provide a protective effect against muscle damage induced by subsequent longer-duration DW. However, this has never been investigated in previous studies, and it is important to examine the magnitude of its protective effect, if any, since a single-joint eccentric exercise model does not necessarily reflect muscle damage in an exercise in which multi-joint movements are performed [15].

Therefore, the present study investigated whether a prior bout of 5-min DW would confer a protective effect against muscle damage induced by a subsequent bout of 40-min DW performed 1 week later. To induce muscle damage, the same protocol of DW (gradient: -28%, velocity: 5 km/h, load: 10% of body mass, duration: 40 min) as that of our previous study [12] was used. This protocol has been used as a model of DW-induced muscle damage [8, 12]. It should be noted that actual trekking activities are more complex in gradient, and their time and duration could range from a few hours to days [1, 2]. The present study chose 5-min DW as the preconditioning exercise based on our pilot study showing that 5-min DW performed by untrained young men (n = 5) did not decrease knee extensor torque and increase plasma CK activity. The present study also included another group that performed 5-min level walking (LW) 1 week before 40-min DW. LW is the most primary form of physical activity, and involves less magnitude of eccentric contractions when compared to DW [16], so its protective effect against subsequent DW was assumed to be minimum. We hypothesized that 5-min DW, but not 5-min LW, would reduce the magnitude of muscle damage induced by 40-min DW performed 1 week later.

## Materials and methods

### Subjects

The sample size was estimated using the data from our previous study [12] in which the effect of 20-min DW on subsequent 40-min DW was examined. On the basis of an α level of 0.05 and a power (1 − β) of 0.8, with a potential 10% difference in maximal voluntary isometric contraction (MVC) torque of the knee extensors at 24 h after 40-min DW between the conditions with and without the 5-min DW, it was found that 12 participants per group would be sufficient. Thus, 36 healthy young men were recruited and placed to either a control or one of the two preconditioning walking groups (PRE-DW or PRE-LW, n = 12/group). In this process, the baseline physical characteristics (i.e. height, body mass, MVC torque) were intentionally matched among the groups to minimize potential confounding factors. No significant (*P* > 0.05) differences in the mean (± SD) age (23.5 ± 4.5 years), height (1.68 ± 0.07 m), body mass (64.7 ± 6.4 kg), and MVC torque of the knee extensors (197.5 ± 12.1 Nm) were evident among the groups. None of the subjects had been involved in any type of systematic (≥ 30 min/day, ≥ 2 days/week) resistance, aerobic, or flexibility training program, and had experienced mountain trekking and/or downhill walking (other than those encountered in daily activities) in the past 6 months. This study was approved by the ethics committee of the National Institute of Fitness and Sports in Kanoya (7–49) and was conducted in accordance with the policy statement regarding the use of human participants by the Declaration of Helsinki. The participants visited the laboratory, and were fully informed about the purpose, procedures, and possible risks involved in the study, and provided a written informed consent before participation in the study.

### DW and LW

DW and LW were performed on a treadmill with the slope of -28% (DW) and 0% (LW), respectively, with the velocity of 5 km/h, and a load of 10% body mass added to a typical hiking backpack [12]. Participants were instructed to walk at their most comfortable stride length and frequency, and not to change it throughout each trial and across trials. The velocity of 5 km/h was fast for walking, but no participant reported that it was too fast to walk naturally. The PRE-DW and PRE-LW groups performed 5-min DW (~110-m descent: vertical length) and 5-min LW, respectively, under the condition shown above 1 week before 40-min DW (~900-m descent), since we [12] showed some protective effect of 20-min DW (~450-m descent) on 40-min DW that was performed 1 week later. The control group performed 40-min DW without performing 5-min DW or LW. All sessions were supervised by one of the authors, and performed at the temperature of 20–25°C and relative humidity of 40–60%. Participants wore their own sports shoes and clothes, and wore the same shoes across the trials. Participants were not allowed to wear any compression garments during each trial, and instructed not to perform any unfamiliar activities, and not to have any interventions that could affect the recovery such as massage, icing, and nutritional supplementations during the experimental period.

### Muscle damage markers

**Fig 1:**
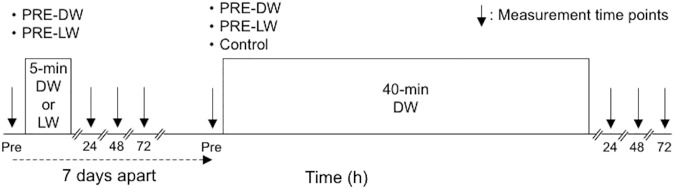
Schematic diagram of the experimental design. The measurements were taken before and 24 h after 5-min DW and 5-min LW, and before and 24, 48, and 72 h after 40-min DW.

The indirect markers of muscle damage consisted of isometric MVC torque of the knee extensors, plasma CK activity, and muscle soreness assessed by a visual analogue scale (VAS), which were the same as those used in our previous study [12]. These were measured before and 24 h after 5-min DW and 5-min LW, and before, 24, 48 and 72 h after 40-min DW (Fig 1).

#### MVC torque of the knee extensors

MVC knee extension torque of the right leg was measured using a custom-made dynamometer in the same manner previously reported [12]. Briefly, each participant sat in an adjustable chair with a support for the back while the hip and knee joints were kept at 90° (anatomical position: 0°). Although it has been reported that peak isometric knee extension torque occurs at ~80° [17, 18], the posture used in this study (knee and hip joints both at 90°) has been shown to detect changes in MVC knee extension torque induced by DW accurately [8, 12, 19]. After performing 2–4 submaximal (50–80% MVC) contractions, the participants performed two MVCs with a 1-min rest between trials. The participants were asked to produce maximal force of the knee extensors for 5 s. Additional trials were performed if the difference in the peak torque of the two trials was more than 10%. The trial with the highest peak torque was used for further analysis. The day-to-day (separated by 2–3 days) reproducibility of the MVC measurement was examined on 6 healthy men in our pilot study. The coefficient of variation (CV) and the intraclass correlation coefficient (*r*) for this variable were 6.5% and 0.85, respectively.

#### Plasma CK activity

Plasma CK activity was measured by a Reflotron (Reflotron plus system; F. Hoffmann-La Roche, Switzerland) loading a 30-μL whole blood sample collected using a capillary pipette from a finger prick to a CK test strip (Reflotron CK Test; F. Hoffmann-La Roche, Switzerland). A pilot study showed that the test–retest reliability of the 2 blood samples taken at the same time was high (CV < 1%), thus only one blood sample was collected during each measurement for each participant. The normal reference range for this method is 24–195 U/L based on the company’s documentation (http://www.cobas.com/content/dam/cobas_com/pdf/product/Reflotron-Plus-Sprint-system/Brochuere%20Reflotron.pdf)

#### Muscle soreness

The magnitude of muscle soreness at the anterior thigh perceived during squat was assessed using a VAS consisting of a 100-mm line representing “no pain” at one end (0 mm) and “unbearable pain” at the other (100 mm). Participants were instructed to perform a squat movement (knee flexed until at ~90°) with the feet shoulder-width apart and the arms crossed over the chest, from which they rated the level of soreness by drawing a line on the scale provided [8]. Based on the Universal Pain Assessment Tool (http://pronursingservice.com), which is often used in a clinical setting, the severity of muscle soreness was categorized as no (VAS: < 10 mm), mild (10–29 mm), moderate (30–69 mm), and severe (> 70 mm).

### Statistical analysis

The normality of the data was checked and subsequently confirmed by a Shapiro-Wilk’s test for all variables except for pre-exercise VAS, which was 0 for all participants. Thus, we did not perform between-time comparisons for VAS changes. Changes in MVC torque and plasma CK activity following 5-min DW and 5-min LW for the two groups were compared by a two-way (2 groups x 2 time points) repeated measures analysis of variance (ANOVA), and those following 40-min DW were compared amongst the three groups by a two-way (3 groups x 4 time points) repeated measures ANOVA. When a significant interaction effect was found for the changes in MVC torque and plasma CK activity after 40-min DW, a one-way ANOVA (4 time points) followed by Dunnett's post hoc test were performed to compare changes from the pre-value for each group, and a one-way ANOVA and Tukey post-hoc test were performed for the comparison between the groups for each time point. For muscle soreness (VAS), we did not perform ANOVA (because pre-exercise VAS was 0 for all participants), and comparisons between the groups were made at each time point after 5-min DW or 5-min LW (at 24 h) by an unpaired t-test, and after 40-min DW (at 24, 48, and 72 h) by a one-way ANOVA and Tukey post-hoc test. As indices of effect size, *Cohen’s d* (for *t* tests and post hoc comparisons) and partial *η*^2^ (for ANOVA) values were also calculated. Sphericity was checked by Mauchly’s test in ANOVA and *P* values were modified with greenhouse-geisser correction when necessary. All data were analyzed using SPSS software (version 23.0, IBM Corp, USA), and are presented as mean ± SD.

## Results

### Changes in muscle damage markers following 5-min DW and 5-min LW

No significant changes in any of the variables were evident following 5-min DW and 5-min LW (Table 1). A two-way ANOVA did not find any significant main or interaction effect on MVC torque (group: *P* = 0.922, partial *η*^2^ < 0.001; time: *P* = 0.586, partial *η*^2^ = 0.014; interaction: *P* = 0.110, partial *η*^2^ = 0.112) and plasma CK activity (*P* = 0.513, partial *η*^2^ = 0.020; *P* = 0.381, partial *η*^2^ = 0.035; *P* = 0.906, partial *η*^2^ = 0.001) between 5-min DW and 5-min LW. Muscle soreness after 5-min DW and 5-min LW was 4.2 ± 4.9 mm and 2.1 ± 2.8 mm, respectively, without a group-difference (*P* = 0.215, *Cohen’s d* = 0.53).

**Table 1:** Changes in muscle damage markers following 5-min downhill walking and 5-min level walking.

		PRE-DW	PRE-LW
MVC (Nm)	Pre	201.1 ± 22.2	194.7 ± 30.6
	24 h	193.9 ± 20.9	198.3 ± 30.0
CK (U/L)	Pre	132.3 ± 50.0	146.7 ± 36.5
	24 h	140.5 ± 95.4	157.3 ± 54.2
VAS (mm)	Pre	0.0 ± 0.0	0.0 ± 0.0
	24 h	4.2 ± 4.9	2.1 ± 2.8

Changes (mean ± SD, n = 12/group) in maximal voluntary isometric contraction torque of the knee extensors (MVC), plasma creatine kinase activity (CK), and muscle soreness assessed by a 100 mm visual analog scale (VAS) before (Pre) and 24 hours after 5-min downhill or level walking for the preconditioning downhill walking (PRE-DW) and level walking (PRE-LW) groups, respectively.

### Changes in muscle damage markers following 40-min DW

**Fig 2:**
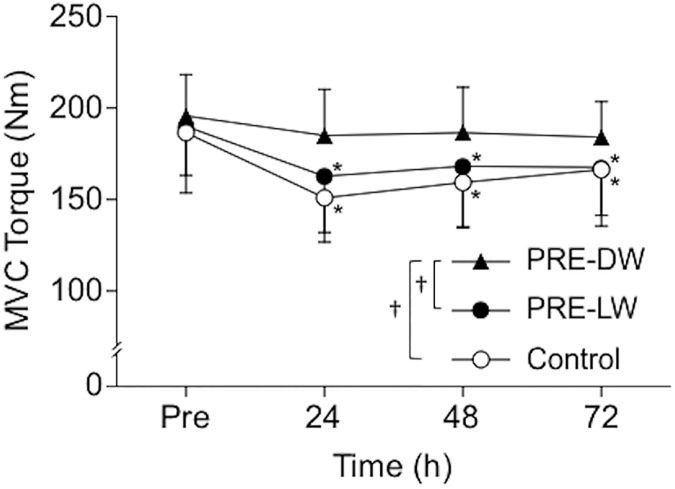
Changes in maximal strength following 40-min downhill walking. Changes in maximal voluntary isometric contraction (MVC) torque of the knee extensors before (pre), and 24, 48, and 72 hours after 40-min downhill walking for the preconditioning downhill walking (PRE-DW, closed triangle), preconditioning level walking (PRE-LW, closed circle), and control (open circle) groups. Values are means ± SDs. An asterisk (*) indicates a significant (*P* < 0.05) difference from the pre-value (baseline). A cross mark (†) indicates a significant (*P* < 0.05) difference between groups based on a two-way and subsequent one-way ANOVA.

Changes in MVC torque before and after 40-min DW are shown in Fig 2. Significant (*P* = 0.048, partial *η*^2^ = 0.118) interaction was found in the MVC torque changes. MVC torque significantly decreased for the PRE-LW (*P* < 0.002, *Cohen’s d* = 0.76–0.95) and control groups (*P* < 0.001, *Cohen’s d* = 0.69–1.24), and did not recover to the baseline at 72 h post-DW for these groups. On the other hand, PRE-DW group did not show any significant changes after 40-min DW (*P* > 0.10, *Cohen’s d* = 0.39–0.55).

**Fig 3:**
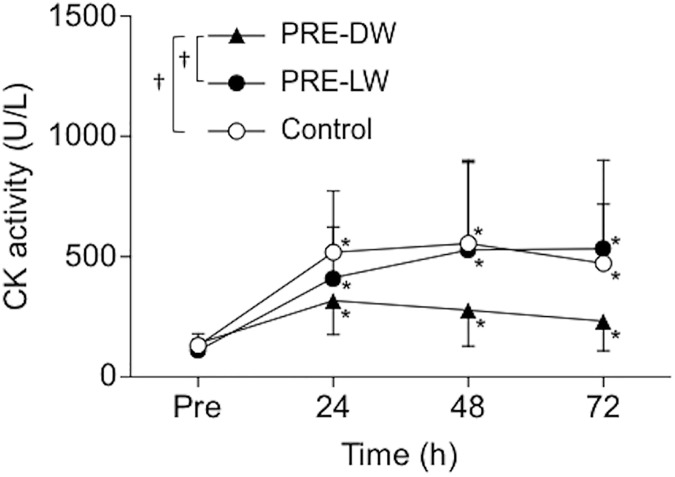
Changes in plasma creatine kinase activity following 40-min downhill walking. Changes in plasma creatine kinase (CK) activity before (pre), and 24, 48, and 72 hours after 40-min downhill walking for the preconditioning downhill walking (PRE-DW, closed triangle), preconditioning level walking (PRE-LW, closed circle), and control (open circle) groups. Values are means ± SDs. An asterisk (*) indicates a significant (*P* < 0.05) difference from the pre-value (baseline). A cross mark (†) indicates a significant (*P* < 0.05) difference between groups based on a two-way and subsequent one-way ANOVA.

Fig 3 shows changes in plasma CK activity before and after 40-min DW. Plasma CK activity increased for all groups (PRE-DW: *P* < 0.03, *Cohen’s d* = 0.99–1.69; PRE-LW: P < 0.03, *Cohen’s d* = 1.57–1.99; control: P < 0.001, *Cohen’s d* = 1.77–2.11), but the magnitude of increase was smaller for the PRE-DW than the other two groups. A significant difference between the PRE-DW and PRE-LW was found at 72 h post-DW (*P* = 0.01, *Cohen’s d* = 1.10).

**Fig 4:**
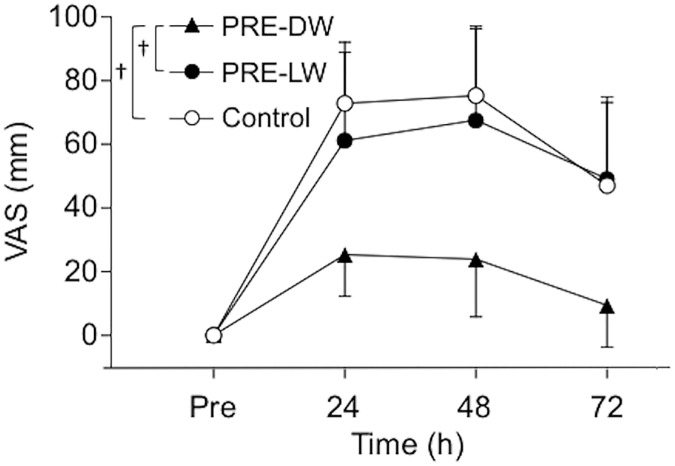
Changes in muscle soreness following 40-min downhill walking. Changes in muscle soreness of the anterior thigh assessed by a visual analog scale (VAS) before (pre), and 24, 48, and 72 hours after 40-min downhill walking for the preconditioning downhill walking (PRE-DW, closed triangle), preconditioning level walking (PRE-LW, closed circle), and control (open circle) groups. Values are means ± SDs. A cross mark (†) indicates a significant (*P* < 0.05) difference between groups based on a one-way ANOVA.

Fig 4 shows changes in muscle soreness (VAS) before and after 40-min DW. Muscle soreness developed in all groups, but was smaller for the PRE-DW than the other two groups at 24, 48 and 72 h post-DW (*P* < 0.001, *Cohen’s d* = 1.51–3.34).

## Discussion

Neither 5-min DW nor 5-min LW induced any symptoms of muscle damage (Table 1). However, the changes in the muscle damage markers following 40-min DW were significantly smaller for the PRE-DW group than the control and PRE-LW groups (Figs 2–4). This supported the hypothesis that 5-min DW, but not 5-min LW, would confer protective effects on changes in muscle damage markers after 40-min DW. The present results were in line with the findings of the studies reporting that low-intensity eccentric exercise in the absence of symptoms of muscle damage conferred a protective effect against subsequent potentially damaging eccentric exercise of the elbow flexors in young men [13] and the knee extensors in older men [14] using a single-joint model. The present study is the first to show that this was also the case for DW, which is a more practical, multi-joint eccentric exercise model, and represents possible muscle damage in trekking [3].

The 40-min DW in the control group showed symptoms of muscle damage. MVC torque decreased by 19% at 24 h post-DW and remained lower than the baseline for 72 h post-DW (Fig 2), and plasma CK activity significantly increased and did not recover at 72 h after 40-min DW (Fig 3). The peak VAS of the muscle soreness exceeded 75 mm (Fig 4), which was classified as severe pain (> 70 mm) according to the Universal Pain Assessment Tool (http://pronursingservice.com). It appears that these changes are typical following long-duration DW (e.g. 40 min) without any preconditioning exercise in a similar protocol, as shown by our previous study [12] and others [20, 21]. Howatson et al. [3] reported significant changes in these variables (e.g. peak strength reduction: ~10%, peak CK: ~350 U/L, peak soreness: ~70/200 mm) after an actual trekking (Snowdon in UK, ascent/descent = 756 m, taking ~4.5 h including scheduled stops) in recreationally active men and women. On the other hand, the magnitude of the changes in the variables after 40-min DW in the PRE-DW group was smaller than that of the control group by 49% for MVC torque, 47% for plasma CK activity, and 66% for soreness in their peak values. These suggest that the 5-min DW attenuated the magnitude of muscle damage induced by 40-min DW. We [12] showed that 20-min DW provided a protective effect on subsequent 40-min DW by 60% for MVC torque, 55% for plasma CK activity, and 84% for soreness based on their peak changes. This indicates that the 5-min DW was slightly less effective than the 20-min DW for attenuating indices of muscle damage after 40-min DW. However, it should be noted that the 5-min DW that did not result in any changes in indirect markers of muscle damage still conferred the protective effect close to the 20-min DW that induced some indications of muscle damage. In the present study and most of the previous studies, no examinations of muscle tissue were performed. Thus, it is not known whether an absence of symptoms of muscle damage indicates no histological signs of muscle damage. However, it appears that muscle damage symptoms are not required for a protective effect to be conferred.

There was no group difference between the control and PRE-LW groups in any of the muscle damage markers after the 40-min DW (Figs 2–4). This indicates that a prior bout of LW did not provide any protective effect against subsequent damaging DW. This is reasonable considering the fact that LW involves less magnitude of eccentric contractions than DW does [16]. It is unknown whether a prior bout of uphill walking confers a protection against DW-induced muscle damage. We did not set an intervention group that performed a prior bout of 5-min uphill walking, because 1) it would be unpractical to perform uphill walking as a preconditioning exercise if one’s goal is to prepare oneself against DW-induced muscle damage, and 2) none of the participants in our pilot study (n = 4) could complete 5-min uphill walking under the same condition as the DW and LW (i.e. velocity: 5km/h, load: 10%, and slope: +28% as compared to -28% for DW) due to the high demand on the cardiovascular system [22]. It seems unlikely that 5-min uphill walking produces protective effect on 40-min DW as 5-min LW did not.

The present study does not provide any data regarding the underpinning mechanisms of the protective effect conferred by the 5-min DW, thus only speculation can be made. McHugh [10] in his review paper suggested a combination of neural, mechanical, and cellular adaptations as possible mechanisms underpinning the repeated bout effect. This may be applied to the protective effect induced by the 5-min DW. For example, a previous study [23] using electromyography showed that fast twitch fibers were preferentially recruited during an initial eccentric exercise bout, but more involvement of slow twitch fibers, which are more resilient to eccentric contraction-induced muscle damage, were observed during the subsequent bout. It has also been reported that the amount of fascicle strain measured by ultrasound during eccentric cycling was smaller in the second than the first bout [24]. Up-regulation of reactive oxidative species [25] and heat shock proteins [26] could also play a role in the protective mechanisms, but it is not known whether these changes occur after 5-min DW. It is interesting to investigate whether these adaptations are induced by eccentric exercise that does not result in any symptoms of muscle damage. Lima and Denadai [9] pointed out in their recent review paper that the protective mechanisms would be different between eccentric exercise inducing some damage versus no damage. They speculated that eccentric exercises resulting in some damage would lead to an addition of new sarcomeres in series and/or strengthening of extracellular matrix, but this would be less likely after non-damaging exercise [9]. Further research is needed to investigate the mechanisms underpinning the protective effect conferred by non-damaging exercise including DW.

This study provides some important practical implication for individuals who are planning to go mountain trekking for the first time or with a long interval from a previous exposure. The present study clearly demonstrated that performing relatively short-duration (e.g. 5-min), non-symptomatic DW attenuated the magnitude of muscle damage following potentially damaging long-duration (e.g. 40-min) DW performed 1 week later. Thus, to reduce or avoid muscle damage potentially induced by DW, it is advisable to perform short-duration, low-intensity DW prior to longer-duration DW within a week. Since such protective effect has been shown to be cumulative [19], it is recommended that short-duration DW be performed several times before potentially damaging DW. It is not known from the present study whether repeated bouts of short-duration DW (e.g. 5-min) attenuate muscle damage in actual trekking (e.g. 6 hours). It may be better to gradually increase the duration of DW over weeks before trekking (e.g. 5 min, 10 min, 20 min, 40 min). Since it was shown in the present study that 5-min DW was effective for reducing muscle damage symptoms after 40-min DW (the exercise duration is 8 times longer), it is interesting to investigate further if 40-min DW is effective for 5-h DW, for example. The descending distance (vertical length) was ~110 m for the 5-min DW in this study, thus it may be that using stairs for a similar distance or duration is an alternative, if there is no access to a treadmill to perform DW. It is highly possible that performing preconditioning exercise reduces eccentric exercise-induced muscle damage and a risk of accidents during mountain trekking.

## Conclusion

This study showed that 5-min DW and 5-min LW caused no indication of muscle damage, but only 5-min DW significantly attenuated muscle damage following subsequent 40-min DW performed 1 week later. This suggests that even non-damaging, short-duration DW is effective in providing a strong protective effect against potentially damaging DW performed within a week. It appears that performing small volume of eccentric contractions is crucial in preparation for DW during trekking.
